# The “No ARSA” Sign: A Novel Method of Prenatal Screening for Aberrant Right Subclavian Artery

**DOI:** 10.3390/jcm9082658

**Published:** 2020-08-17

**Authors:** Eran Kassif, Abraham Tsur, Shir Shust-Barequet, Oshrat Raviv, Anya Kushnir, Samar Abu Snenh, Reuven Achiron, Shali Mazaki-Tovi, Boaz Weisz, Yishay Salem, Tal Weissbach

**Affiliations:** 1 Departments of Obstetrics and Gynecology, Sheba Medical Center, Tel Hashomer, Sackler School of Medicine, Tel Aviv University, Tel Aviv 69978, Israel; eran.kassif@gmail.com (E.K.); avitsur@gmail.com (A.T.); shirshust@gmail.com (S.S.-B.); anyakush@gmail.com (A.K.); samarabusnenh33@gmail.com (S.A.S.); reuvenachiron@gmail.com (R.A.); shalimazaki@gmail.com (S.M.-T.); boazmd2010@gmail.com (B.W.); 2 Departments of Obstetrics and Gynecology, Meir Medical Center, Kfar Saba, Sackler School of Medicine, Tel Aviv University, Tel Aviv 69978, Israel; oshratraviv@gmail.com; 3 Pediatric Cardiology Unit, Sheba Medical Center, Tel Hashomer, Sackler School of Medicine, Tel Aviv University, Tel Aviv 69978, Israel

**Keywords:** brachiocephalic artery, right subclavian artery, aberrant right subclavian artery, prenatal screening, aortic arch anomalies

## Abstract

An aberrant right subclavian artery (ARSA) can be overlooked by the conventional method as described by Chaoui et al., due to acoustic shadowing. The aim of this study was to evaluate the feasibility and accuracy of a novel screening method for ARSA by demonstrating the brachiocephalic artery bifurcation, referred to as the “No ARSA” sign. A prospective study conducted at a tertiary care center between 2018 and 2019 included unselected pregnant patients at a median gestational age of 15.1 (14.2–22.1; IQR (inter-quartile range)) weeks, who had been referred for a routine or targeted anomaly scan. All participants were scanned for the presence or absence of ARSA using both the conventional and the novel “No ARSA” methods for validation purposes. A total of 226 unselected patients were enrolled in the study. The “No ARSA” sign was visualized in 218 fetuses (96.5%). In the remaining 8 cases (3.5%), the “No ARSA” sign was not demonstrated. In these fetuses, an ARSA was visualized by the conventional method. The new method exhibited 100% feasibility and was in complete agreement with the conventional method. Intra- and inter-observer agreement was excellent (κ = 1). The results of the study suggest that the “No ARSA” sign is an efficient and reliable screening tool for ARSA.

## 1. Introduction

The prenatal diagnosis of an aberrant right subclavian artery (ARSA) in fetuses with Down syndrome was first reported in 2005 by Chaoui et al. [1]. Since then, it was reported in a variety of genetic aberrations and was found to be associated with cardiac and extra cardiac anomalies [1,2,3,4,5,6,7,8,9]. In postnatal life it is usually asymptomatic but on occasion can cause symptoms secondary to tracheal and esophageal compression [10]. The most common symptoms include dysphagia, cough and stridor. Of these, dysphagia is probably the most recognized symptom, and gives rise to the term, “dysphagia lusoria” [11,12,13,14]. 

The brachiocephalic artery (BCA) is the first artery to emerge from the aortic arch. Shortly after its origin, it bifurcates to the right subclavian artery (RSA), which courses posterolateral towards the right arm, and the right carotid artery (RCA). The RCA is a direct continuation of the brachiocephalic artery, coursing cranially through the fetal neck towards the fetal head.

In 1.23–2.2% of the cadaveric population, the right subclavian artery arises aberrantly from the distal aortic arch and courses behind the trachea and esophagus to supply the right arm [15,16]. In these patients, the RCA emerges as a single vessel directly from the aortic arch and does not bifurcate.

The aim of this study was to evaluate the feasibility and accuracy of a novel screening method for ARSA by demonstrating the BCA bifurcation to the RCA and the RSA, the “No ARSA” sign.

## 2. Materials and Methods

This was a cross-sectional prospective study conducted at a tertiary care center between the years 2018 and 2019. Unselected pregnant patients referred for a routine or targeted anomaly scan were included in the study. All participants were consented and scanned by one of the two participating scanners (E.K. and T.W.), each with different scanning experience (20 and 3 years, respectively). The presence or absence of ARSA was specifically determined using two methods: 1. the conventional method and 2. the novel “No ARSA” method. The conventional method was considered “gold standard”, as it has been previously validated in numerous studies. For quality control and validation purposes, a third of the images and clips were randomly selected and examined by a pediatric cardiologist (Y.S.). For intra-observer agreement assessment, the BCA bifurcation was interrogated twice during the scan by the same sonographer in 20 patients. For inter-observer agreement assessment, a separate study was performed, including 20 unselected patients that were consented and scanned by both scanners (E.K. and T.W.) in a blinded fashion.

### 2.1. Description of the “No ARSA” Method

**Figure 1:**
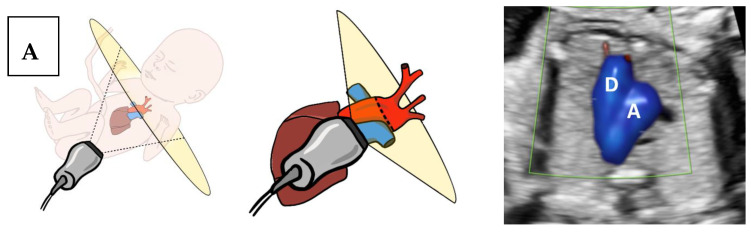

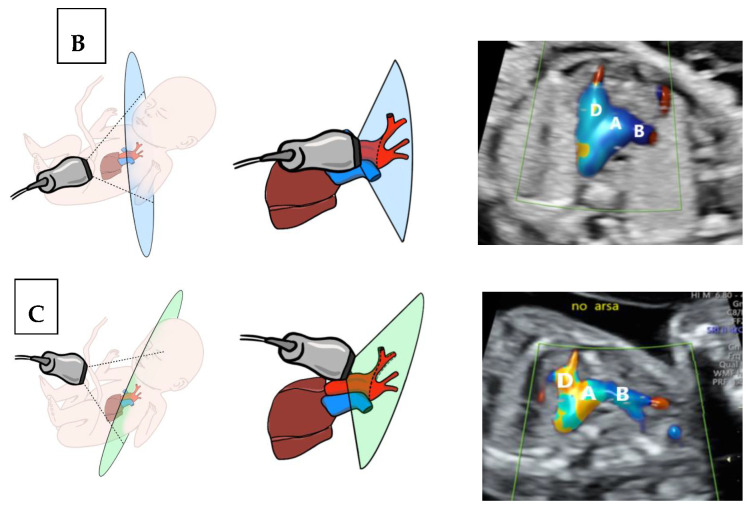
“No ARSA” plane acquisition: The brachiocephalic artery arises from the aortic arch and crosses anterior to the trachea and towards the right side of the fetus. The angle of insonation to acquire the corresponding plane to demonstrate (**A**) three-vessel trachea (3VT) view (**B**) brachiocephalic artery origin (**C**) brachiocephalic artery bifurcation, the “No ARSA” sign. ARSA, aberrant right subclavian artery; D, Ductus arteriosus; A, Aortic arch; B, Brachiocephalic artery.

This method is based on the demonstration of the brachiocephalic artery bifurcation to the RCA and the RSA (the “No ARSA” sign). In order to demonstrate the “No ARSA” sign, the fetus should ideally be in a supine position with a slight right-sided tilt. The three-vessel trachea view is then obtained in the axial plane, as described by Yagel et al. [17] (Figure 1A). It includes the ductus arteriosus branching from the pulmonary trunk, a transverse section of the aortic arch, located to the right of the ductus arteriosus, and finally, cross sections of the superior vena cava and trachea, located to the right of the aorta. From the 3VT (three-vessel trachea) plane, the BCA is visualized arising from the aortic arch (Figure 1B) by slightly rotating the transducer towards the sagittal plane. 

**Figure 2:**
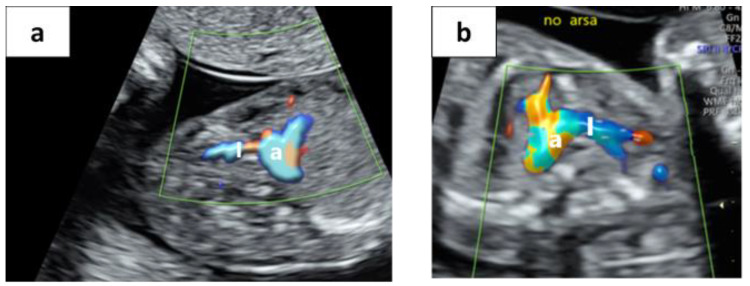
**The “No ARSA” sign.** The brachiocephalic artery (I) arises from the aortic arch and bifurcates into the RCA (right carotid artery), which courses as a continuum of the brachiocephalic artery cranially and the RSA (right subclavian artery) coursing posterolateral (**a**) at 14.3 weeks and (**b**) at 21.2 weeks.

By further rotation of the transducer towards the sagittal plane, the brachiocephalic artery’s long axis is exposed with its bifurcation to the right carotid artery and the right subclavian artery (Figure 1C and Figure 2 and Video S1). Once the BCA bifurcation (the “No ARSA” sign) is demonstrated, ARSA is ruled out. 

**Figure 3:**
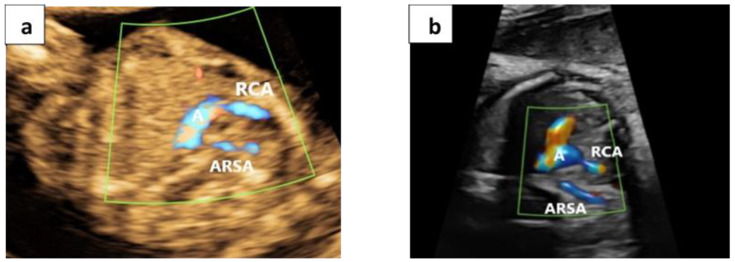
Negative "No ARSA" sign: The RCA emerging as a single vessel from the aortic arch with no visible bifurcation. Simultaneously, an ARSA is visualized coursing behind the trachea towards the right arm (**a**) at 14.2 weeks and (**b**) at 32.5 weeks. RCA, Right carotid artery; ARSA, Aberrant right subclavian artery. A, Aortic arch.

If the bifurcation is not visible, the single vessel is then followed up to the neck to confirm that it is the right carotid artery and to ensure the bifurcation was not overlooked (Video S2). Once the bifurcation of the first vessel of the aortic arch is ascertained to be absent, an ARSA is suspected. In order to validate this method, all fetuses were also scanned using the conventional method described by Chaoui et al. [1] (Figure 3). 

Examinations were performed transabdominally or transvaginally, depending on gestational age, using a Voluson E10 ultrasound machine (GE Healthcare, Milwaukee, WI, USA) with either an abdominal RM6C 2–6 MHz convex probe or a vaginal RIC 6–12 MHz probe, as appropriate. Generally, a transvaginal approach was used at 12–15.6 weeks of gestation and a transabdominal approach from 16 weeks and over. 

Maternal demographic information, medical history, fetal anomaly scan results, prenatal genetic evaluation and postnatal outcomes were collected. A comparison of maternal and fetal parameters was performed between the “No ARSA” and ARSA groups. 

The study was approved by the Institutional Review Board (5345-18-SMC). 

### 2.2. Statistical Analysis

Normality of the data was tested using the Shapiro-Wilk or Kolmogorov-Smirnov tests. Data are presented as median and inter-quartile range (IQR). Comparison between unrelated variables was conducted with Student’s *t*-test or a Mann–Whitney U test, as appropriate. The chi-square and Fisher’s exact tests were used for comparison between categorical variables. Cohen’s kappa was used for inter- and intra-observer agreement. Significance was accepted at *p* < 0.05. Statistical analyses were conducted using the IBM Statistical Package for the Social Sciences (IBM SPSS v.23; IBM Corporation Inc., Armonk, NY, USA, https://www.ibm.com/analytics/spss-statistics-software). 

## 3. Results

A total of 226 unselected patients were enrolled in the study and scanned, either on a routine early (12–16 weeks; *n* = 126) anomaly scan, a routine mid-trimester (19–26 weeks; *n* = 88) anomaly scan or during a targeted anomaly scan for various conditions and at different weeks of gestation (*n* = 12). 

We were able to demonstrate the “No ARSA” sign in 218 fetuses (96.5%) and at various gestational ages (Figure 2). In all these fetuses, an ARSA was also ruled out using the conventional method, without false negative cases. In eight fetuses (3.5%) the course of the first vessel arising from the aortic arch was followed up to the neck without an apparent bifurcation. In all these cases, an ARSA was confirmed using the conventional method. In some of these cases, we were able to demonstrate both the ARSA and RCA arising separately from the aortic arch in the designated “No ARSA” plane (Figure 3 and Video S3). We were able to correctly confirm or refute the BCA bifurcation in 100% of cases compared to the “gold standard” conventional method.

### Intra- and Inter-Observer Agreement

Both intra- and inter-observer agreement levels were excellent (κ = 1 for both). The inter-observer assessment was performed in a separate study which included 20 unselected patients that were scanned by both investigators in a blinded fashion (E.K. and T.W.). None of the patients were found to have a fetus with an ARSA, using both methods. 

The intra-observer assessment was an integral part of the study. In 20 cases, the BCA was interrogated twice during the same scan by the same investigator. There was complete agreement in all 20 cases. 

The demographic, medical history and pregnancy characteristics of the study group are presented in Table 1. We compared the “No ARSA” group to the proven ARSA group. There were no statistically significant differences among the groups regarding maternal and pregnancy characteristics. All ARSA cases were isolated. Amniocentesis was carried out in five of the eight cases, all with a normal microarray. The diagnosis of an ARSA was performed at 23.4 weeks at the latest. The demonstration of the “No ARSA” sign was also feasible at term.

## 4. Discussion

The prenatal detection of an ARSA is of importance because of its association with Down syndrome and a variety of other genetic syndromes, especially when non-isolated [1,2,3,5,6,7,8,9,18,19,20,21,22,23]. Furthermore, ARSA itself can occasionally cause aggravating symptoms due to compression of the esophagus and trachea [11,12,13,14]. 

There is a discrepancy in the reported prevalence of ARSA in cadaveric studies in comparison to that in prenatal ultrasound studies, possibly explained by insufficient prenatal detection of this condition. Two large-scale cadaveric studies have reported a prevalence of 1.23–2.2% [15,16], while prenatal ultrasound studies in unselected populations have reported a prevalence of only 0.4% [4,24]. A low ARSA prevalence was also found in additional prenatal studies [7,18,25]. All of these studies screened patients using the conventional method, as it was the only ARSA screening method published and validated during the time of these studies. Indeed, the lower reported prenatal prevalence of ARSA, compared to that found among the cadaveric population, might be due to an underdiagnosis of this condition prenatally.

Furthermore, the conventional method of ARSA detection is not always feasible. A study assessing the conventional method at the first and second trimester scans reported a feasibility of 85.3% and 98%, respectively, with an overall feasibility of 94% [26]. The authors suggested that feasibility was dependent on fetal crown-rump length and maternal body mass index. Of note, 4 ARSA cases were missed on the first trimester scan. Most of the scans were performed transabdominally. 

On the other hand, our study showed a feasibility of 100% in both the first and second trimesters, using a transvaginal approach up to 16 weeks of gestation and a transabdominal approach at 16 weeks and over. The experience of the scanners was not an obstacle, as one of the scanners was less experienced, with only 3 years of ultrasound training. All of the cases that demonstrated a “No ARSA” sign were confirmed to be normal on the “gold standard” conventional method without false negative cases. Moreover, the results of the intra- and inter-observer assessment conducted showed the "No ARSA" to be a reproducible and reliable sign. 

A complementary method for ARSA screening is essential for two main reasons. First, by relying solely on the conventional method, cases with ARSA can be missed due to acoustic shadow cast by the sternum, clavicle and rib cage, obscuring the retro-tracheal field. In contrast, the plane of interest in the "No ARSA" method is closer to the probe and less obscured by acoustic shadows, as the BCA bifurcation is located more superficially in the fetal body, in the right anterior part of the lower fetal neck. Secondly, without an alternative confirmatory screening method, once an ARSA is deemed absent by the conventional method, it is impossible to acknowledge if it is truly nonexistent or erroneously overlooked.

Our results show a 3.5% rate of ARSA, clearly higher than reported by previous fetal and cadaveric studies. This high rate is probably related to the high-risk population scanned at our tertiary referral center. All eight cases of ARSA in our study were isolated. In five of the cases, a microarray was performed, with normal results in all. This observation is in agreement with previous studies that have found that chromosomal abnormalities are rare among cases with an isolated ARSA [20,21,22,23,25,27]. On the other hand, there are contradicting reports, with data pointing to an association between isolated ARSA and aneuploidy [4,7,8,9,19].

### 4.1. Important Anatomical Landmarks

The knowledge of the spatial anatomy of the aortic arch and its branches in relation to adjacent structures facilitates the demonstration of the “No ARSA” sign, which is straightforward once the 3VT view is acquired. The ascending aorta arises from the left ventricle, positioned anteriorly and slightly to the right of the trachea. The aortic arch crosses the midline anterior to the trachea and over the left main bronchus towards the left side of the trachea and, from this point, it courses caudally alongside the thoracic esophagus. The fetal trachea has a characteristic appearance on ultrasound with bright echogenic walls and a sonolucent fluid-filled lumen. It is an important anatomical landmark of the midline. 

The BCA is the first arterial trunk to emerge from the aortic arch (Figure 1). It arises from the arch anteriorly to the trachea and after a short cranio-lateral course towards the right, it bifurcates into the RSA, coursing posteriorly towards the right shoulder and the RCA, then cranially through the right side of the fetal neck. By interrogating the aortic component of the 3VT plane, it is easy to detect the origin of the BCA and follow its short path to the point of bifurcation to the RSA and RCA and appreciate their different anatomical paths. Once the bifurcation of the BCA cannot be visualized, a vigorous attempt to demonstrate an ARSA by the conventional method should be pursued. Furthermore, the first branching artery should be followed to confirm it is in fact the RCA. 

### 4.2. Distinguishing Variants of the Left Aortic Arch

When scanning the normally left aortic arch, one should keep in mind possible variants. Most malformations of the aortic arch can be explained by the hypothetical double arch theory described by Edwards [28,29]. The ARSA is the result of erroneous regression of the left aortic arch segment between the right subclavian and right carotid arteries. Other variants of the left aortic arch exist, such as separate origins of common carotid arteries, subclavian arteries and vertebral arteries directly from the arch, or a common trunk giving rise to the right BCA and left common carotid artery, or a separate origin of the left vertebral artery proximal to the left subclavian artery [30,31]. All these variants will present with an abnormal number of vessels arising either from the arch itself or from its first arising vessel. Therefore, if the first vessel of the aortic arch bifurcates into two vessels, one coursing cranially and the other coursing posterolaterally, then the scanner can be confident that an ARSA was not overlooked due to a variant of the arch vessels.

We believe that this method is simple and easy to acquire. By implementing this straightforward method routinely in the low-risk general population, we may increase the prenatal detection rate of an ARSA and thereby improve our prenatal counseling. This is especially important in non-isolated cases. 

The strengths of our study are its prospective nature and the inclusion of an unselected population, minimizing various bias effects. In addition, the present study includes a relatively wide gestational age range, which extends its internal validity across all three trimesters. The limitations of our study should also be acknowledged. The setting of the study is a tertiary reference center, which may account for the relatively high prevalence of ARSA in this series. Nevertheless, we refrain from using any statistical tests that are affected by disease prevalence (e.g., positive and negative predictive value). In addition, the level of expertise of tertiary care center sonographers is high and might have contributed to a high feasibility rate. This might not represent the feasibility that would have been achieved at a community-based center. 

## 5. Conclusions

We present a novel, simple and feasible screening method for ARSA. The new method shows good performance when compared to the conventional method. The results of the present study suggest it to be an efficient and reliable screening tool for ARSA. Routine implementation of the “No ARSA” method might enhance the prenatal detection of ARSA.

## Figures and Tables

**Table 1:** Characteristics of study group.

Variables	Total *n* = 226	No ARSA *n* = 218	ARSA *n* = 8	*p* Value
Maternal Age (y)	34 (30–36)	34 (30–36)	33 (30–36.5)	0.47
Nulliparity	36.4% (80/220)	37.7% (80/212)	0%	0.053
Pre-pregnancy BMI (kg/m^2^)	24 (23.2–37.1)	21.4 (20.7–24.2)	21 (20–22)	0.76
Pre-pregnancy BMI ≥ 25	18.9% (38/201)	19.2% (37/193)	12.5% (1/8)	0.64
IVF Pregnancy (%)	7.9% (17/215)	8.2% (17/207)	0%	0.4
High-Risk Pregnancy * (%)	26.8% (59/220)	27.4% (58/212)	12.5% (1/8)	0.7
Gestational Age at Exam (w.d)	15.1 (14.2–22.1)	15.1(14.2–22.1)	18.5 (14.1–22.5)	0.8
Gestational Age Range at Exam (w.d)	12.3–37.5	12.3–37.5	13.6–23.4	
EFW at Exam (gr)	135 (98–505)	135 (98–504)	301.5 (98.5–564)	0.97
EFW Range at Exam (gr)	63–3048	63–3048	93–633	
Male Gender	53.8% (70/130)	54.4% (68/125)	40% (2/5)	0.66
Other Anomalies ** (%)	12.8% (29/226)	13.3% (29/218)	0%	0.3
Chromosomal Microarray Abnormality *** (%)	2.6% (2/78)	2.7% (2/73)	0% (0/5)	1

BMI, body mass index; IVF, in vitro fertilization; EFW, estimated fetal weight; ARSA, aberrant right subclavian artery. Data are given as median (interquartile range) or percentage (*n*/N). *, diabetes, previous preterm birth, advanced maternal age, etc. **, 10 cardiovascular, 9 renal, 3 gastrointestinal, 3 neural axis, 3 facial, 1 musculoskeletal; ***, trisomy 21, 9p 24.3 duplication.

## Supplementary Materials

The following are available online at https://www.mdpi.com/2077-0383/9/8/2658/s1, Video S1: Demonstration of the “No ARSA” sign, Video S2: Negative “No ARSA” sign: following the RCA from the aortic arch to the fetal head in an axial plane, Video S3: Demonstration of both the ARSA and RCA arising separately from the aortic arch in the designated “No ARSA” plane.
